# Nutrient restriction protects against valve interstitial cell calcification by upregulating ubiquitin mediated proteolysis

**DOI:** 10.3389/fcvm.2025.1586775

**Published:** 2025-07-21

**Authors:** K. Phadwal, Q. Tang, D. Kurian, X Tan, W. P. Cawthorn, V. E. MacRae

**Affiliations:** 1 The Roslin Institute and Royal (Dick) School of Veterinary Studies, University of Edinburgh, Midlothian, United Kingdom; 2 University/BHF Centre for Cardiovascular Science, The Queen’s Medical Research Institute, Edinburgh Bioquarter, University of Edinburgh, Edinburgh, United Kingdom; 3 School of Life Sciences, Faculty of Science and Engineering, Anglia Ruskin University, Cambridge, United Kingdom

**Keywords:** nutrient restriction, calcific aortic valve disease, ubiquitin mediated proteolysis, Cullin-2, SILAC

## Abstract

**Introduction:**

Calcific aortic valve disease (CAVD) is a common and progressive valvular heart disease characterised by the pathological calcification of valve interstitial cells (VICs). Current clinical treatments, such as surgical valve replacement and transcatheter valve implantation, are invasive and do not target the underlying molecular mechanisms of calcification. Emerging evidence suggests that metabolic interventions may modulate cellular calcification processes. In this study, we investigated the potential of nutrient restriction (NR) as a non-invasive strategy to mitigate VIC calcification, with a particular focus on the role of the ubiquitin-proteasome system (UPS).

**Methods:**

Primary rat valvular interstitial cells (RVICs) were cultured and subjected to in vitro calcification using calcium- and phosphate-enriched media. Nutrient restriction was induced by incubating cells in Hank's Balanced Salt Solution (HBSS). Calcification was assessed by quantifying calcium deposition and osteogenic marker expression. To explore the underlying molecular changes, a stable isotope labelling by amino acids in cell culture (SILAC)-based proteomic analysis was performed. The role of the UPS was further examined using pharmacological inhibition with MG132 and siRNA-mediated knockdown of key UPS components, including Cullin-2 (Cul2) and Ubiquitin-conjugating enzyme E2 H (Ube2H).

**Results:**

Nutrient restriction significantly downregulated the expression of osteogenic markers and reduced calcium deposition in RVICs. SILAC-based proteomics revealed the upregulation of multiple components of the UPS in nutrient-restricted cells. Notably, Cul2 and Ube2H were identified as potential key mediators of the anti-calcification effects observed. Inhibition of the proteasome with MG132 exacerbated calcification, while knockdown of Cul2 using siRNA increased osteogenic marker expression and calcium deposition, indicating the essential role of Cul2 in modulating VIC calcification under nutrient-restricted conditions.

**Conclusion:**

This study demonstrates that nutrient restriction effectively attenuates VIC calcification through the modulation of the ubiquitin-proteasome system. The protective role of UPS components, particularly Cul2 and Ube2H, suggests that targeting this pathway could represent a novel therapeutic approach for the management of CAVD. These findings also raise the possibility of employing dietary or metabolic interventions as non-invasive strategies to prevent or delay valve calcification.

## Introduction

Aortic stenosis (AS) is the most prevalent valvular heart disease, with a reported prevalence of 10% for severe AS in adults aged 80 years and older (1). Degenerative calcific aortic valve disease (CAVD), predominantly age-related, constitutes the most common etiology of AS in the elderly, affecting over 25% of individuals over 65 years of age (2). The pathophysiology of CAVD shares significant parallels with physiological bone mineralization. Valvular interstitial cells (VICs), the predominant cell type within the aortic valve, play a central role in the progression of CAVD. Prior research has demonstrated that VICs can undergo osteogenic differentiation, acquiring a bone-like phenotype accompanied by increased protein expression of osteogenic markers such as RUNX family transcription factor 2 (RUNX2) (3), osteocalcin (OCN) (4), bone sialoprotein (BSP) (5) and osterix (6).

Current treatment options for CAVD primarily involve surgical valve replacement or transcatheter aortic valve implantation (TAVI) with prosthetic valves (7). However, these interventions are invasive and carry high risks. Additionally, prosthetic valves have limited durability, ultimately succumbing to structural degeneration and calcification. Therapeutic strategies targeting aging or extending healthspan are notably absent from the current standard of care for CAVD. While lifestyle modifications, including smoking cessation and increased physical activity, have been integrated into the management of cardiovascular risk factors, these interventions do not directly address the aging process itself. Notably, over a century of aging research has identified dietary interventions as among the most effective approaches for extending lifespan and healthspan across various organisms (8). Nutritional strategies aimed at healthspan extension therefore represent a promising avenue for addressing CAVD without introducing adverse effects.

NR, including regimens such as fasting or ketogenic diets, holds considerable potential for exerting protective cardiovascular effects (9). While NR protocols vary widely—ranging from time-restricted feeding to prolonged fasting or diets restricted in specific nutrients—several common systemic responses to NR have been observed. These include reductions in circulating levels of glucose, insulin, insulin-like growth factor 1 (IGF-1), growth hormone, leptin and adrenaline, alongside increases in ketone bodies, IGF-binding protein 1 (IGFBP1), fibroblast growth factor 21 (FGF21), cortisol, adiponectin and glucagon (10). At the cellular level, NR suppresses growth pathways such as mechanistic target of rapamycin (mTOR) and mitogen-activated protein kinases (MAPK), activates the master energy sensor AMP-activated protein kinase (AMPK), and induces autophagy (11, 12). In the present study, we demonstrate, for the first time, the potential of nutrient restriction (NR) to mitigate calcification in VICs, thereby highlighting NR as a promising non-invasive intervention for preventing VIC calcification.

## Materials and methods

### RVIC culture and calcification

Rat valvular interstitial cells (RVICs) were cultured in DMEM/F-12 growth medium (Gibco), supplemented with 10% fetal bovine serum (FBS) and 1% gentamycin (Gibco), and seeded in 12-well plates at a density of 3 × 10^4^ cells per well. Calcification was induced as previously described (13). Cells were maintained until they reached 70%–80% confluence, at which point they were exposed to either control medium supplemented with 1.05 mM calcium (Ca) and 0.95 mM phosphate (Pi) or calcifying media containing 2.5 mM Ca and 2.7 mM Pi. Cultures were incubated for up to 72 h in a humidified environment at 5% CO₂.

### *In vitro* nutrient deprivation

To induce nutrient deprivation, cultured cells were maintained in DMEM/F-12 growth medium (Gibco) until they reached the desired confluence (typically 70%–80%). Subsequently, the growth medium was aspirated, and the cells were gently rinsed with pre-warmed phosphate-buffered saline (PBS) to remove residual nutrients. The cells were then incubated in Hank's Balanced Salt Solution (HBSS), supplemented with 2.5 mM calcium (Ca) and 2.7 mM phosphate (Pi), at 37°C in a humidified atmosphere containing 5% CO₂ for 24–48 h. Following the nutrient deprivation period, the HBSS was replaced with appropriate culture medium for downstream treatments or experimental analysis.

### Cell viability assay

Cells were seeded into a microplate at a density of 3 × 10^4^ cells per well and allowed to adhere overnight. Subsequently, the Alamar Blue reagent was added to the culture medium at a concentration constituting 10% of the total volume, as previously described (14). The plate was incubated at 37°C in a humidified atmosphere with 5% CO₂ for up to 4 h, during which metabolically active cells reduced the reagent, resulting in a detectable colorimetric change. Absorbance was measured at wavelengths of 570 nm and 600 nm. The results were then analyzed to assess cell viability across samples, with the degree of reagent reduction serving as an indicator of metabolic activity.

### MG132 treatment

To induce UPS inhibition, cultured cells were maintained in DMEM/F-12 growth medium (Gibco) until they reached the desired confluence (typically 70%–80%). The cells were then incubated with the 20S proteosome inhibitor MG132 (Cayman) for 48 h (50 or 100 nm), supplemented with 2.5 mM Ca and 2.7 mM Pi, at 37°C in a humidified atmosphere containing 5% CO₂. Following MG132 treatment, cells were collected for further experimental analysis.

### Alizarin red staining

Cells were cultured in plates and subjected to calcification treatment as previously described (13). After removing the culture medium, the cell monolayer was gently rinsed with PBS and fixed in 10% neutral-buffered formalin (NBF) for 15 min at room temperature (RT). Following fixation, the cells were washed twice with PBS to remove residual fixative. The monolayer was then stained with 2% Alizarin S solution (pH 4.2, 500 µl per well) for 10 min at RT to detect calcium deposition. Excess staining solution was discarded, and the cells were washed thoroughly three times with distilled water to remove unbound dye. The plates were allowed to air-dry completely before imaging.

### Calcium assay

Calcium deposition was quantified according to the established protocol (13). Briefly, RVICs were washed with PBS and subsequently incubated in 0.6 N hydrochloric acid (HCl) at 4°C for 24 h to solubilize deposited calcium. The liberated calcium was quantified colorimetrically using a commercially available assay kit based on its stable interaction with ơ-Cresolphthalein (Randox Laboratories). The measured calcium content was subsequently normalized to the total protein concentration of each sample, determined using the DC protein assay (Bio-Rad Laboratories).

### RNA interference (RNAi) treatment

RVICs at approximately 70% confluence were utilized for siRNA transfection studies following an established protocol (15). For each transfection, 6 μl (60 ρM) siRNA targeting Cul2 (Santa Cruz) or non-targeting scrambled control (SC) siRNA (Santa Cruz) was used. The cells were incubated at 37°C in Opti-MEM medium (Gibco) containing the respective siRNA and Lipofectamine 3,000 (Thermo Fisher) for up to 7 h. Subsequently, the transfection medium was replaced with complete growth medium, and the cells were cultured for an additional 72 h to facilitate siRNA-mediated gene silencing. Following this period, the transfected RVICs were cultured for 24 h in either DMEM or HBSS, both supplemented with 2.7 mM calcium and 2.5 mM phosphate. Subsequently, the cells were collected for downstream analyses.

### SILAC labelling and quantitative proteomics of RVICs

RVICs were cultured over six passages in either “light” DMEM/F-12 R0K0 medium (DC Biosciences, LM038) or “heavy” DMEM/F-12 R10K8 medium (DC Biosciences, LM040), as previously described (16). Both media were supplemented with SILAC-dialyzed FBS (DC Biosciences, DS1002) to ensure proper isotopic labelling. Following the sixth passage, RVICs labelled with “light” isotopes were cultured in DMEM supplemented with calcium and phosphate (DMEM-CaPi), while RVICs labelled with “heavy” isotopes were incubated in HBSS with calcium and phosphate (HBSS-CaPi) for 24 h. After treatment, cells were trypsinized, and cell pellets were washed twice with cold PBS prior to sample preparation for quantitative mass spectrometry (MS).

Cell pellets were resuspended in 10% trifluoroethanol (pH 8.0) and subjected to gentle vortexing for 1 h to ensure solubilization. Proteins were reduced with 5 mM dithiothreitol (DTT) and alkylated with 10 mM iodoacetamide before digestion with sequencing-grade modified trypsin (Promega). The resulting peptide mixtures were centrifuged at 800 × g to pellet any insoluble material, and the supernatant containing digested peptides was collected. The peptides were cleaned using StageTips to remove impurities.

Quantitative proteomics was performed using nanoflow liquid chromatography-tandem mass spectrometry (nanoLC-MS/MS) on a micrOTOF-II mass spectrometer (Bruker, Germany) coupled to an RSLCnano LC system (Thermo). Tryptic peptides were first delivered to a trap column at a flow rate of 20 μl/min with 100% solvent A (0.1% formic acid in LC-MS grade water) for 4 min of loading and washing. Peptides were then transferred to an analytical column and separated at 300 nl/min using a 60-min gradient from 7% to 35% solvent B (0.1% formic acid in acetonitrile). Eluted peptides were electrosprayed directly into the mass spectrometer for MS and MS/MS analysis in a data-dependent acquisition mode. MS scans were performed over the m/z range of 300–2,000, followed by MS/MS scans of the six most intense ions. Rolling collision energy was applied for fragmentation based on precursor ion mass, and dynamic exclusion was set at 30 s to avoid repeated fragmentation of the same ions.

Raw spectral data were processed using DataAnalysis software (Bruker), and imported into ProteinScape 3.1 server (Bruker), where the spectral data were searched against the Uniprot rat sequence database (containing 22,367 entries) using Mascot 2.4 (Matrix Science). Search parameters included a peptide precursor ion mass tolerance of 25 ppm and a fragment ion mass tolerance of 0.05 Da. Peptide charge states were set to 2+ and 3+, with carbamidomethylation of cysteine as a fixed modification and oxidation of methionine, Label:13C(6)15N(4) of Arginine and Label:13C(6)15N(2) of Lysine, as variable modifications. Peptide identifications were filtered to achieve a false discovery rate (FDR) of <1% by incorporating a decoy database search. SILAC quantification was performed by using WARPLC plugin on Proteinscape 3.1 software to integrate extracted ion chromatogram of every precursor. Peptide ratios were normalized based on setting overall peptide median ratio at one, which corrects for unequal protein sampling and a coefficient of variability of peptide ratios were also determined for each quantified protein.

### Immunoblotting

Immunoblotting was performed as previously reported (17, 18). Protein lysates were prepared using radioimmunoprecipitation assay (RIPA) buffer supplemented with the Halt Protease Inhibitor Cocktail (Thermo Fisher Scientific). The protein concentration of the lysates was quantified using the Pierce BCA Protein Assay Kit (Thermo Fisher Scientific). Samples were mixed with 4× loading buffer, boiled and resolved on a 4%–12% Bis-Tris gel (Invitrogen) via sodium dodecyl sulfate–polyacrylamide gel electrophoresis (SDS-PAGE). Proteins were subsequently transferred onto polyvinylidene difluoride (PVDF) membranes (Thermo Fisher Scientific). Membranes were blocked for 1 h using 5% skimmed milk in phosphate-buffered saline with 0.1% Tween-20 (PBST) and incubated overnight at 4°C with primary antibodies diluted 1:1000 in 5% skimmed milk. Primary antibodies included those against Runx2 (Abcam, ab236639), Cul2 (Proteintech, 10981-2-AP), Ube2h (Proteintech, 15685-1-AP), OCN (Proteintech, 23418-1-AP), Osterix (Proteintech, 28694-1-AP), β-actin (Proteintech, 66009-1-Ig) and bone sialoprotein (Invitrogen, PA5-79425). Following washing, membranes were incubated for 1 h with secondary antibodies. Horseradish peroxidase (HRP)-conjugated goat anti-rabbit IgG (Dako, P0449) was employed. Blots were then visualized using the GeneGnome imaging system (Syngene).

### ATP measurement

Intracellular ATP levels were measured using a commericial ATP Assay Kit (ab83355, Abcam) according to the manufacturer's instructions. Briefly, cells were washed with cold PBS, lysed in ATP Assay Buffer, and centrifuged at 13,000 × g for 5 min at 4°C to remove insoluble material. Supernatants were collected and incubated with the ATP reaction mix containing ATP Converter, Developer, and ATP Probe in a 1:1 ratio. After 30 min of incubation at room temperature in the dark, the signal was measured using a microplate reader at 570 nm (colorimetric). ATP concentrations were calculated based on a standard curve generated using known concentrations of ATP.

### Statistical analysis

All data are presented as the mean ± standard error of the mean (SEM). Statistical analyses were performed using either a paired *t*-test or one-way analysis of variance (ANOVA) followed by Tukey's *post hoc* test, depending on the experimental design. All computations were conducted with GraphPad Prism software, and statistical significance was defined as a *p*-value less than 0.05. Significance levels were indicated as follows: *p < 0.05, **p < 0.01, and ***p < 0.001. The sample size (N) corresponds to the number of independent cultures evaluated.

## Results

### Nutrient deprivation inhibits calcification in RVICS

**Figure 1:**
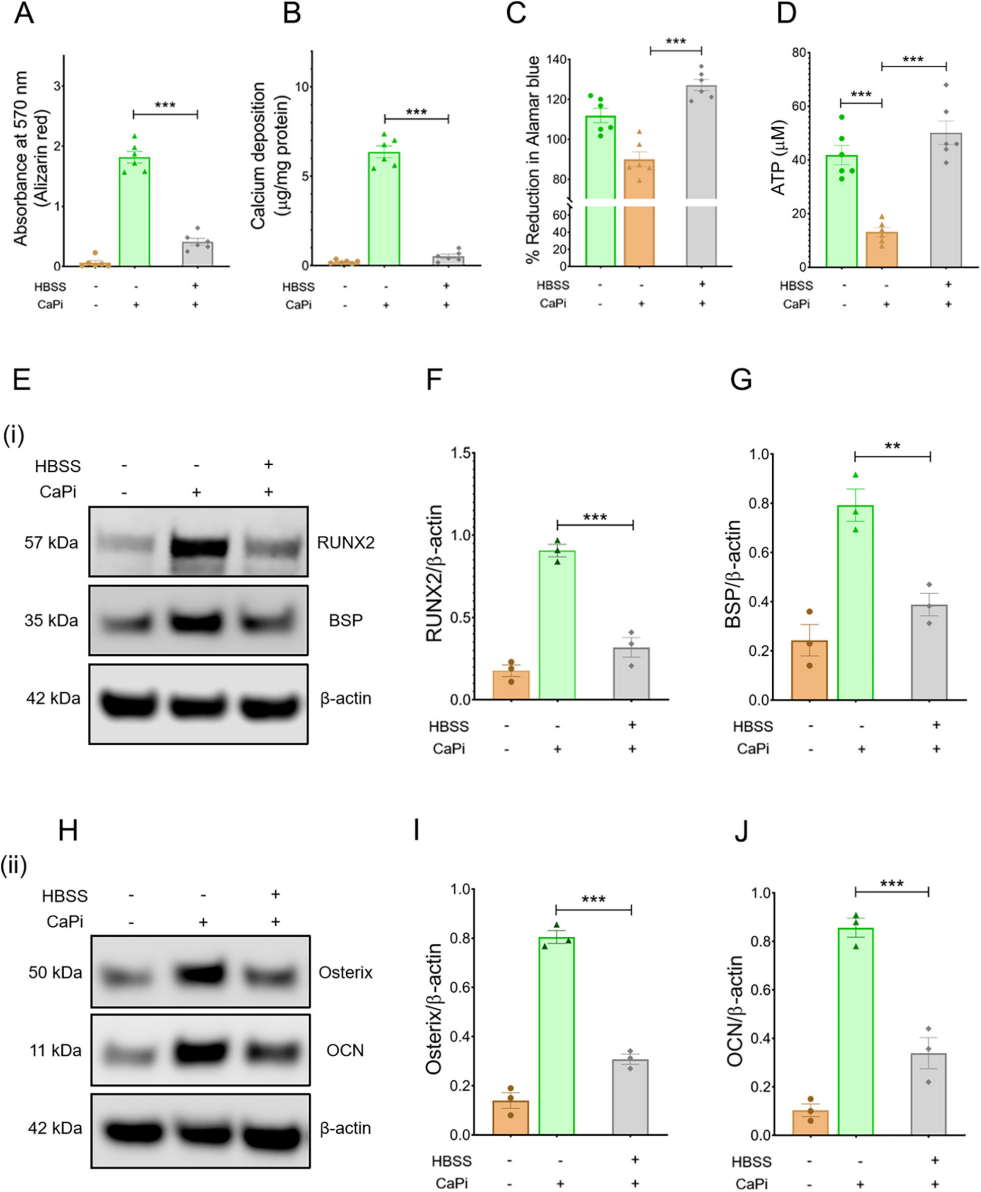
Calcification is reduced in nutrient-starved RVICS. **(A)** Quantification of alizarin red staining for RVICs cultured either with complete DMEM media or HBSS supplemented with or without 2.7Ca/2.5Pi for 48 h (*N* = 6). **(B)** Quantification of calcium deposition in RVICs cultured with complete DMEM media or HBSS in the presence of 2.7Ca/2.5Pi for 48 h (*n* = 6). **(C)** Cell viability of RVICs cultured in complete DMEM media in the presence or absence of 2.7Ca/2.5Pi and HBSS in the presence of 2.7Ca/2.5Pi for 48 h using the Alamar blue assay (*n* = 6). **(D)** ATP measurement of RVICs cultured with or without complete DMEM media in the presence or absence of 2.7Ca/2.5Pi and HBSS in the presence of 2.7Ca/2.5Pi for 48 h using the Alamar blue assay (*n* = 6). **(E)** Representative images of immunoblots for RUNX2, BSP and β-actin in RVICs cultured with or without complete DMEM media or HBSS supplemented with 2.7Ca/2.5Pi for 48 h. **(F,G)** The graphs show the ratios of RUNX2 and BSP to β-actin. **(H)** Representative images of immunoblots for osterix, OCN and β-actin in RVICs cultured with or without complete DMEM media or HBSS supplemented with 2.7Ca/2.5Pi for 48 h. **(I,J)** The graphs show the ratios of osterix and OCN to β-actin. Equal amounts of total protein were loaded in each lane, and all blots were processed in parallel under identical experimental conditions to ensure comparability. *p < 0.05, **p < 0.01, ***p < 0.001.

To assess the impact of NR on RVIC calcification, calcification was evaluated in RVICs cultured in either complete DMEM media or HBSS, an established model of nutrient deprivation (19), in the presence of 2.7 mM Ca and 2.5 mM Pi. Nutrient deprivation significantly attenuated calcification in RVICs, as demonstrated by reduced alizarin red staining and diminished calcium deposition (Figures 1A,B). Cell viability assessment revealed that RVICs displayed enhanced viability after 48 h of HBSS treatment (Figure 1C). Consistently, intracellular ATP levels were elevated in the HBSS-treated group compared to the calcified group, further supporting the observation of enhanced metabolic activity (Figure 1D). Additionally, nutrient deprivation resulted in a marked downregulation of osteogenic marker protein expression in RVICs, including RUNX2, osterix, BSP and OCN (Figures 1E–J). Together these findings demonstrate that nutrient deprivation effectively reduces calcification in RVICs.

### Ubiquitin proteasome pathway is enriched in nutrient-deprived RVICs

**Figure 2:**
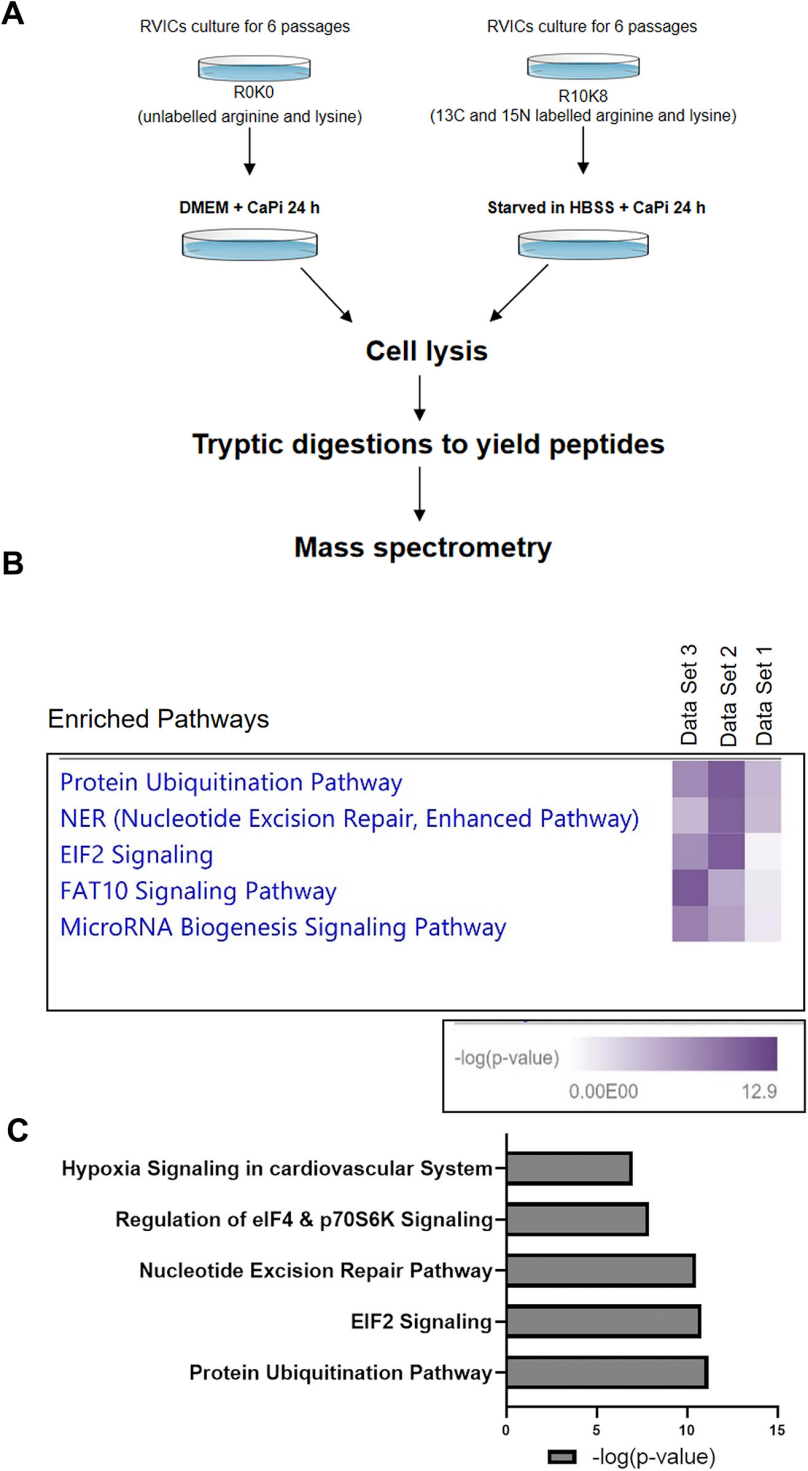
Proteomic analysis of RVICs cultured in DMEM media or HBSS supplemented with 2.7Ca/2.5Pi. **(A)** Schematic diagram of the experimental design. **(B)** Combined IPA analysis of MS shows enrichment of protein ubiquitination pathway in all three of the independent data sets (*n* = 3). **(C)** Representative graph shows enrichment of protein ubiquitination pathway with HBSS treatment, as indicated by the mean -log10(*P*) value derived from three independent datasets.

To further investigate the mechanisms by which nutrient deprivation attenuates calcification, RVICs were cultured for six passages in DMEM R0K0 and DMEM R10K8 SILAC media, followed by a 24 h calcification period in either DMEM or HBSS supplemented with 2.7 mM Ca^2^⁺ and 2.5 mM Pi. The cells were subsequently harvested for protein extraction and analyzed using LC-MS/MS (Figure 2A). Integrated ingenuity pathway analysis (IPA) of the proteomic data revealed that, compared to calcification in DMEM, calcification in HBSS enriched the protein ubiquitination pathway, nucleotide excision repair (NER) pathway, and eukaryotic initiation factor 2 (EIF2) signaling across the three experimental samples (Figures 2B,C). Notably, the most significant changes were observed in the protein ubiquitination pathway. These data suggest that the protein ubiquitination pathway represents a novel potential mechanism through which Nutrient deprivation mitigates RVIC calcification.

### Cul2 and Ube2H are increased in nutrient-deprived RVICs

**Figure 3:**
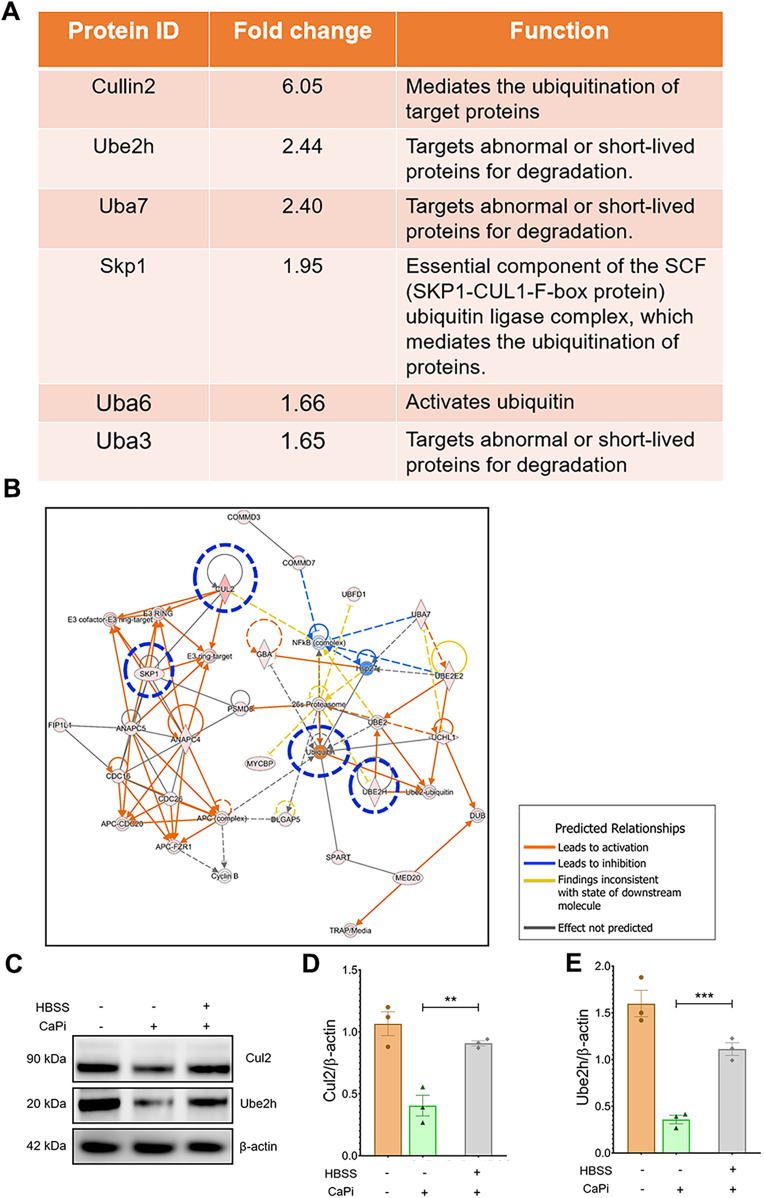
Proteomic analysis shows the upregulated UPS proteins in nutrient-deprived RVICs. **(A)** Fold change and function of proteins involved in ubiquitin proteasome pathway. **(B)** IPA network analysis shows altered activation of key UPS pathway proteins including Cul2, Ube2H, SKP1 and Ubiquitin highlighted in blue circle. Functional interconnections between the proteins are shown by arrows and lines. The blue lines represent direct associations with Annexin VI. Dashed lines represent predicted associations. **(C–E)** Validation and quantification of Cul2 and Ube2h expression by immunoblots. Equal amounts of total protein were loaded in each lane, and all blots were processed in parallel under identical experimental conditions to ensure comparability. *p < 0.05, **p < 0.01, ***p < 0.001.

To further investigate the roles of key proteins within the protein ubiquitination pathway, differentially expressed proteins (DEPs) exhibiting a fold change >1.5 in the HBSS-treated vs. DMEM-treated cells were identified (Figure 3A). Among these, Cul2 and Ube2H displayed the highest fold changes. Predicted protein-protein interactions within the ubiquitination pathway are illustrated in Figure 3B. Consistent with these findings, the upregulation of Cul2 and Ube2H at the protein level was validated by immunoblotting (Figures 3C–E). Collectively, these results indicate that Cul2 and Ube2H may play pivotal roles in the protein ubiquitination pathway in nutrient-deprived RVICs.

### UPS inhibition enhances calcification in RVICS

**Figure 4:**
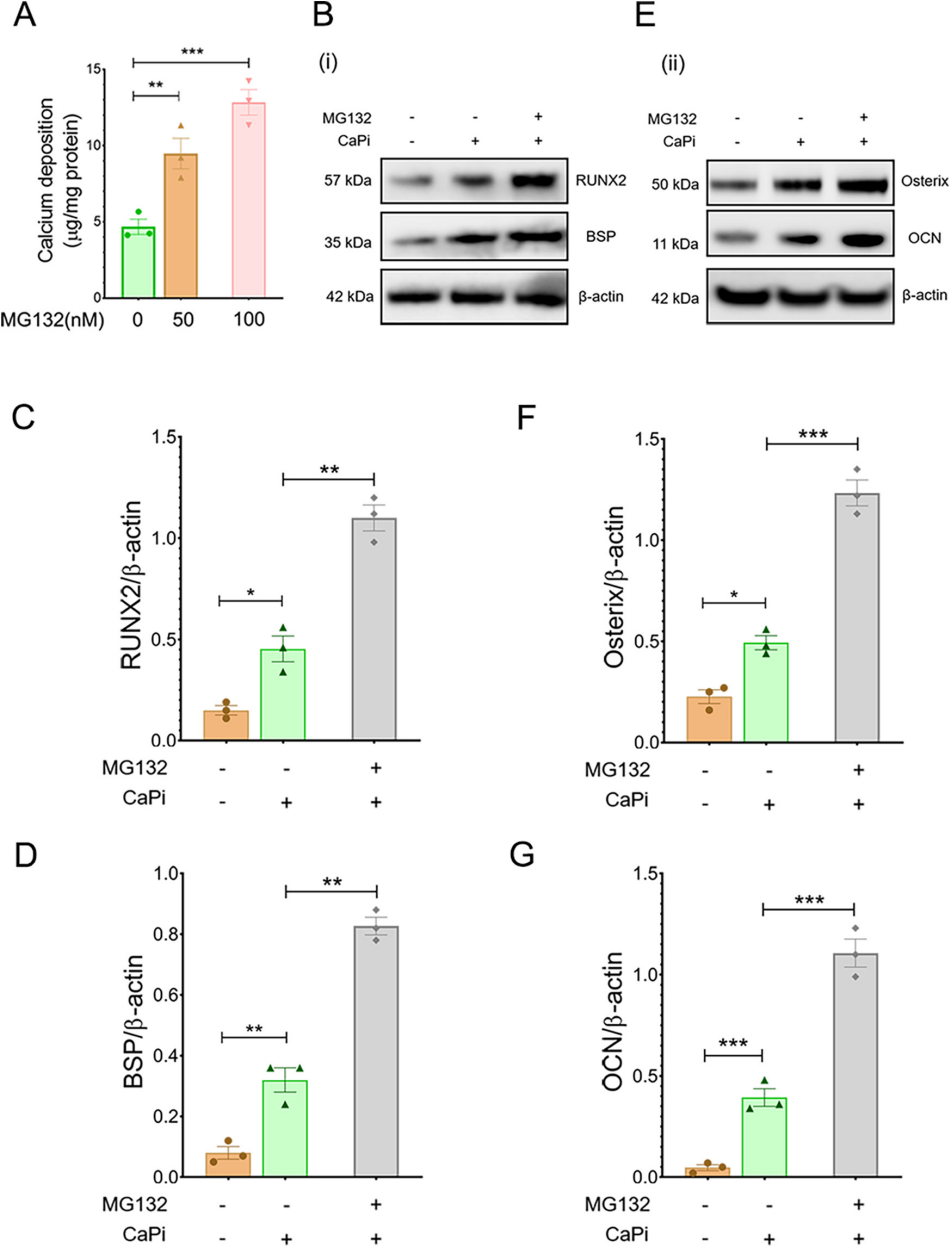
Pharmacological inhibition of Cul2 increases calcification in RVICS. **(A)** Quantification of calcium deposition in RVICs treated with 50 nM or 100 nM MG132 in complete DMEM media supplemented with 2.7Ca/2.5Pi for 48 h (*n* = 3). **(B)** Representative images of immunoblots for RUNX2, BSP and β-actin in RVICs cultured with or without 2.7Ca/2.5Pi supplemented DMEM media in the presence or absence of 100 nM MG132 for 48 h (*n* = 3). **(C,D)** The graphs show the ratios of RUNX2 and BSP to β-actin. **(E)** Representative images of immunoblots for osterix, OCN and β-actin in RVICs cultured with or without 2.7Ca/2.5Pi supplemented DMEM media in the presence or absence of 100 nM MG132 for 48 h (*n* = 3). **(F,G)** The graphs show the ratios of osterix and OCN to β-actin. Equal amounts of total protein were loaded in each lane, and all blots were processed in parallel under identical experimental conditions to ensure comparability. *p < 0.05, **p < 0.01, ***p < 0.001.

**Figure 5:**
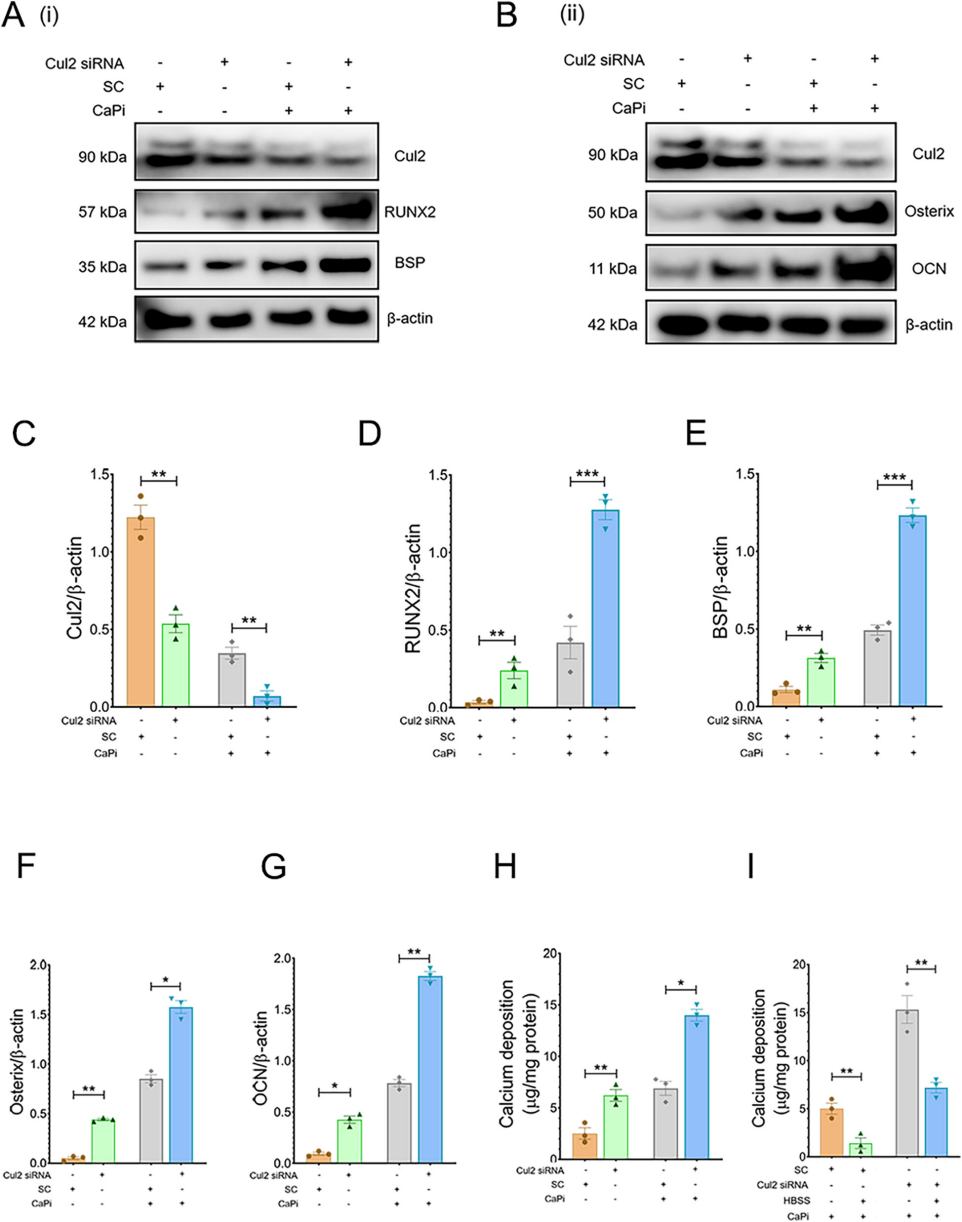
Cul2 knockdown increases calcification in RVICS. **(A,B)** Representative images of immunoblots for RUNX2, osterix, BSP, OCN and β-actin in RVICs transfected with either scrambled control (SC) or Cul2 siRNA for 72 h (*n* = 3) in the presence or abcense of 2.7Ca/2.5Pi. **(C–G)** The graphs show the ratios of Cul2, RUNX2, osterix, BSP and OCN to β-actin. Equal amounts of total protein were loaded in each lane, and all blots were processed in parallel under identical experimental conditions to ensure comparability. **(H)** Quantification of calcium deposition in RVICs transfected with either SC or Cul2 siRNA in the presence or absence of 2.7Ca/2.5Pi for 72 h (*n* = 3). **(I)** Quantification of calcium deposition in RVICs transfected with either SC or Cul2 siRNA for 72 h in 2.7Ca/2.5Pi supplemented DMEM media or HBSS. *p < 0.05, **p < 0.01, ***p < 0.001.

To further assess the role of the ubiquitination pathway in regulating RVIC calcification, RVICs were exposed to MG132, a selective inhibitor of the 20S proteasome. Treatment with MG132 markedly enhanced calcium deposition (Figure 4A) and upregulated the protein expression of osteogenic markers, including RUNX2, osterix, BSP and OCN, in calcified RVICs (Figures 4B–G). Consistent with these findings, silencing Cul2, a key regulator of UPS (20), resulted in increased osteogenic transdifferentiation under both calcifying and non-calcifying conditions, as evidenced by elevated levels of RUNX2, osterix, BSP and OCN (Figures 5A–G) alongside elevated calcium deposition (Figure 5H). However, HBSS treatment alleviated calcification in RVICs both in the presence and absence of Cul2 knockdown (Figure 5I). Collectively, these results indicate that the protein ubiquitination pathway plays a critical regulatory role in RVIC calcification.

## Discussion

CAVD is the most common valvular heart disease worldwide (2). However, there are not presently any pharmaceutical treatments available to impair its progression. NR is therefore an appealing alternative to drug development or repurposing approaches. With growing evidence suggesting widespread beneficial health benefits of NR, this offers an exciting therapeutic option for CAVD. Our data support and extend a body of work proposing that NR can improve metabolic parameters, reduce cardiovascular risk factors and potentially delay the onset of age-related diseases (21). In this study we show for the first time that nutrient deprivation through treatment with HBSS significantly attenuates calcification in VICs. This observation aligns with recent research in hepatocellular carcinoma cells, whereby HBSS-induced *in vitro* nutrient deprivation enhanced the therapeutic response of the targeted cancer drug sorafenib (22).

To investigate the molecular adaptations of calcifying VICs under NR, a comprehensive SILAC analysis was performed. Our analysis revealed a pronounced upregulation of the protein ubiquitination pathway following HBSS treatment, consistent with previous findings suggesting that NR exerts cardioprotective effects through the UPS (23). This is further supported by *in vitro* studies showing that amino acid deprivation activates the UPS in human lung adenocarcinoma epithelial cells (24), as well as mechanistic studies in yeast, which revealed that NR can preserve UPS activity in aged cells (26). Our findings further demonstrate, for the first time, that treatment with MG132, an established proteasome inhibitor (25, 26), exacerbates VIC calcification, thereby highlighting the protective role of protein ubiquitination in mitigating calcification.

Ubiquitination, a highly conserved post-translational modification, involves a three-step enzymatic process mediated by E1 ubiquitin-activating enzymes, E2 ubiquitin-conjugating enzymes and E3 ubiquitin ligases, culminating in the covalent attachment of ubiquitin to substrate proteins (27). Indeed, the UPS system has been recently highlighted as a key contributor to lifespan (28). In the present study, SILAC analysis identified the upregulation of ubiquitin-activating enzymes Uba3, Uba6 and Uba7 in HBSS-treated calcifying VICs. These enzymes are known to activate ubiquitin-like modifiers, including NEDD8 (29), SUMO (30) and ISG15 (31). Indeed, NEDD8 modification and SUMOylation have been recognized as critical regulators of arterial calcification (32, 33). Moreover, we observed increased protein expression levels of Ube2h, an E2 enzyme implicated in the NEDD8 conjugation pathway, further highlighting the significance of ubiquitin-like modifiers in calcification regulation (34). Together these novel findings suggest that ubiquitin-like modifiers could serve as promising therapeutic targets for both arterial and valvular calcification, warranting further investigation to explore their potential clinical application.

Our SILAC analysis revealed a significant upregulation of Cul2 protein levels in calcifying VICs treated with HBSS. Cul2, a member of the Cullin protein family, functions as a scaffold protein that assembles with Elongin B, Elongin C, Rbx1 and various substrate recognition receptors to form Cullin-RING E3 ubiquitin ligase complexes. These complexes play a pivotal role in targeting cellular proteins for ubiquitination-mediated degradation via the 26S proteasome (35). We subsequently employed siRNA-mediated knockdown of Cul2 to investigate the impact of the destabilization of the Cullin-RING E3 ubiquitin ligase complex on VIC calcification and. Notably, Cul2 silencing resulted in the increased expression of osteogenic markers and enhanced calcification of VICs, underscoring the novel protective role of Cul2 in mitigating VIC calcification under nutrient-restricted conditions *in vitro*. To our knowledge this is the first demonstration of an inhibitory effect of Cul2 on the calcification process, in either a physiological or pathological setting.

While proteomic analysis using SILAC provides valuable mechanistic insights into how NR affects calcification, it should be noted that that glucose serves as the prominent energy source for VICs (36). Indeed, nutrient depletion and manipulation of metabolic substrates may impact the viability, metabolism and contractile behaviour of VICs (37), and warrants investigation in future studies.

Recent studies have also demonstrated that NR also produces a range of negative effects that are not yet fully understood. These include reduced wound healing capacity, increased cold sensitivity, and altered bone health (38). Further studies exploring the *in vivo* effects of NR on CAVD and long-term safety data are therefore now required in relevant animal models such as the murine wire-induced aortic valve stenosis model (39).

Neverltheless, our studies highlight the potential for exploring the use of NR mimetics as a potential approach, with candidate compounds including resveratrol, rapamycin and metformin (40). Indeed, recent data has demonstrated that the putative NR mimetic metformin exerts protect effects against VIC calcification, however associated changes in the UPS pathway were not examined in this study (41).

Our findings also highlight E3 ubiquitin ligase downstream target proteins as a novel therapeutic strategy against CAVD. These target proteins include APC/CCdh1 which can suppress the MEK/ERK pathway by targeting the senescence inducer BRAF kinase for degradation (42). Additionally, the RBR type E3 ligase Parkin is important in controlling mitochondrial homeostasis and ROS generation (43). As alterations in senescence and mitochondrial function have been recently proposed to drive vascular calcification (44), future exploration of the role of these downstream targets of E3 ubiquitin ligase would be of great interest.

In summary, our study highlights NR as a promising intervention for preventing VIC calcification, with the ubiquitination pathway emerging as a key mediator of this protective effect. The therapeutic potential of proteasome or E3 ligase activators represents a novel avenue for managing calcific aortic stenosis and warrants further investigation.

## Data Availability

The original contributions presented in the study are included in the article/Supplementary Material, further inquiries can be directed to the corresponding authors.
